# Physician assessments of drug seeking behavior: A mixed methods study

**DOI:** 10.1371/journal.pone.0178690

**Published:** 2017-06-23

**Authors:** Michael A. Fischer, John B. McKinlay, Jeffrey N. Katz, Eric Gerstenberger, Felicia Trachtenberg, Lisa D. Marceau, Lisa C. Welch

**Affiliations:** 1 Division of Pharmacoepidemiology and Pharmacoeconomics, Brigham & Women's Hospital, Harvard Medical School, Boston, Massachusetts, United States of America; 2 New England Research Institutes (NERI) and Division of Medicine, Watertown, Massachusetts, United States of America; 3 Massachusetts General Hospital, Harvard Medical School, Boston, Massachusetts, United States of America; 4 Orthopedic and Arthritis Center for Outcomes Research, Department of Orthopedic Surgery and Division of Rheumatology, Brigham and Women’s Hospital, Harvard Medical School, Boston, Massachusetts, United States of America; 5 Department of Epidemiology, Harvard School of Public Health, Boston, Massachusetts, United States of America; Pennsylvania State University College of Medicine, UNITED STATES

## Abstract

**Background:**

Pain complaints are common, but clinicians are increasingly concerned about overuse of opioid pain medications. This may lead patients with actual pain to be stigmatized as “drug-seeking,” or attempting to obtain medications they do not require medically. We assessed whether patient requests for specific opioid pain medication would lead physicians to classify them as drug-seeking and change management decisions.

**Methods and findings:**

Mixed-methods analysis of interviews with 192 office-based primary care physicians after viewing video vignettes depicting patients presenting with back pain. For each presentation physicians were randomly assigned to see either an active request for a specific medication or a more general request for help with pain. The main outcome was assignment by the physician of “drug-seeking” as a potential diagnosis among patients presenting with back pain. Additional outcomes included other actions the physician would take and whether the physician would prescribe the medication requested.

A potential diagnosis of drug-seeking behavior was included by 21% of physicians seeing a specific request for oxycodone vs. 3% for a general request for help with back pain(p<0.001). In multivariable models an active request was most strongly associated with a physician-assigned diagnosis of drug-seeking behavior(OR 8.10; 95% CI 2.11–31.15;p = 0.002); other major patient and physician characteristics, including gender and race, did not have strong associations with drug-seeking diagnosis. Physicians described short courses of opioid medications as a strategy for managing patients with pain while avoiding opioid overuse.

**Conclusions:**

When patients make a specific request for opioid pain medication, physicians are far more likely to suspect that they are drug-seeking. Physician suspicion of drug-seeking behavior did not vary by patient characteristics, including gender and race. The strategies used to assess patients further varied widely. These findings indicate a need for the development of better clinical tools to support the evaluation and management of patients presenting with pain.

## Introduction

Pain is one of the most common presenting complaints in primary care,[1–3] and clinicians struggle to manage pain appropriately. Awareness that pain is often unrecognized or undertreated[4] has stimulated educational efforts to improve pain management, such as recording pain level as the “fifth vital sign.”[5–7] More recently, however, overprescribing of narcotic pain medications has emerged as a public-health problem. Abuse of narcotics and overdoses related to prescription drugs have increased.[8–11] Clinicians seek to help patients with pain while not contributing to problems of addiction, diversion or overdose risk.[12,13]

Physicians are learning more about the problems with narcotic medications from multiple sources, including mass-media reports, regulatory communications, and first-hand experiences with patients. Physicians hope to avoid prescribing narcotics to patients who seek to obtain them either for diversion or due to addiction.[14,15] These attempts may lead clinicians to label certain patients as “drug-seeking,”[16] with the potential to create stigma for those patients and impair the treatment of pain.

Developing approaches to improve pain treatment without contributing to misuse of narcotic medications will require insights into the thought processes physicians employ when encountering different patients presenting with pain. How physicians make clinical decisions about prescribing pain medications, how they respond to patient requests for pain medication, and how they manage the risk of abuse or diversion of narcotic medications are not well understood. We conducted a mixed-methods study evaluating physician responses to patients presenting with painful conditions and requesting medication for pain, assessing whether they identified patients as drug-seeking and what actions they took based on that assessment.

## Materials and methods

We assessed physician decision making for patients presenting with painful conditions using clinically authentic video-based scenarios involving professional actors portraying an undiagnosed “patient” with symptoms strongly suggesting sciatica. Inserted in the presentation was either an active request for a particular medication or a passive request for pain relief in general. Half of the sciatica vignettes concluded with a specific request for oxycodone, while the other half of the vignettes concluded with a general request for help managing pain, without mentioning a specific medication. The parent study included additional scenarios that did not feature opioid pain medications; those scenarios were not included in the present analysis.[17] The specific active request was as follows:

“My wife/husband had some oxycodone left over from some dental surgery and I took one last night and…I mean, it really worked. I was amazed.”;

We implemented a balanced factorial experimental design to estimate the independent effects of factors and interactions between factors that may affect patient management decisions concerning medication requests. Details of the experimental approach appear elsewhere.[17,18] We examined six main effects: two physician factors(gender, years in practice), and four patient factors(race/ethnicity, gender, socioeconomic status, and request style: active vs. passive request).

Each case was developed with input from physicians who regularly encounter patients with similar conditions. Following their development, two clinical coauthors(JK, MF) along with four independent primary care physicians(PCP) confirmed the clinical accuracy and realism of the presentations.

Six professional actors(male and female of each race—White, Black, Hispanic) were recruited in New York City and directed(under physician supervision) to realistically portray a “patient” presenting to their PCP with the symptoms of sciatica. The same actor/actress portrayed four different patients: two request styles (active vs. passive) and two socioeconomic status levels(lower vs. higher–truck driver vs. sales representative; also expressed by style of dress).

Filmed scenarios have advantages over the use of standardized patients and written scripts because they ensure standardization and permit inclusion of informative nonverbal indicators (e.g. facial grimaces, shifting in discomfort, pointing to specific pain location) and they are now widely used in medical education and for credentialing purposes.[19] Each video-based encounter simulated an interview with a PCP and was approximately 5 minutes in duration, reflecting a typical length of patient history during an office visit.[20]

### Sample and recruitment

Altogether, 3*2^2^ = 12 patient characteristic combinations were produced(race, sex, SES). Each combination was portrayed twice to accommodate the drug request, yielding 24 distinct vignettes for each condition. The two physician factors(gender, physician experience) define four strata. Logistical and cost considerations precluded inclusion(as design variables) of other physician characteristics(such as race/ethnicity) which may influence their prescribing behavior. Within each stratum, 48 participants(physicians) were purposively sampled and randomly assigned to view one of the 24 pairs of vignettes. This constituted two replications of the design, yielding a total sample of 192 physicians.

We recruited licensed PCPs in internal medicine or family practice with over half time spent in clinical care from Illinois, Indiana, Missouri, Massachusetts, New Hampshire, and Rhode Island. Screening telephone calls were conducted to identify eligible subjects and an hour-long, in-person interview was scheduled. Each physician subject provided written informed consent and received a stipend ($200) to partially offset lost revenue and to tangibly acknowledge participation. All study procedures were approved by the New England Research Institute Institutional Review Board.

### Data collection

Immediately after viewing the vignettes, all physician subjects completed a semi-structured interview concerning how they would manage the case, including their diagnoses for the sciatica patient, what medications they would prescribe, what non-medical treatments they would suggest, what additional information they would obtain directly from the patient and what testing they would pursue. Responses were provided by the responding physicians and later coded quantitatively and analyzed statistically.

For the active oxycodone requests only (n = 96), subjects took part in a qualitative interview that included two questions about the vignette patient specifically: “Can you tell me about how you made your decision about medications for the patient in the video? What role did the patient’s request have on your decision?” Answers were provided in a “think-aloud” format, allowing for insights into the cognitive reasoning behind the decision-making processes used by the physicians. Probing encouraged respondents to elaborate in directions they viewed as pertinent. Qualitative interviews (n = 95/96; one respondent did not agree to be recorded) were recorded digitally and transcribed verbatim by a professional transcription company. ATLAS.ti software (ATLAS.ti Scientific Software Development GmbH, Berlin, Germany) facilitated data organization and coding.

### Study outcomes

The pre-specified primary outcome for the overall study was whether physicians reported an intention to prescribe the requested medication, with primary analyses focusing on the impact of active requests, patient characteristics, physician characteristics, and organizational factors on this decision. The results of these analyses have been previously described.[17] Briefly, physicians viewing the active request were significantly more likely to prescribe oxycodone for the sciatica patient(20% vs. 1%), but were not significantly more likely to prescribe any narcotic (66% vs. 55%).

The present analysis focuses on whether physicians expressed concern that patients in the vignettes were motivated by a desire to obtain narcotic medications, often referred to as “drug-seeking behavior.”[16] Quantitative outcome measures included whether drug-seeking was included on the physician’s list of specific diagnoses for the sciatica patient and whether drug-seeking was mentioned as a possibility in qualitative responses. Additional outcomes were actions that physicians would take in reaction to a possible diagnosis of drug-seeking behavior including information-seeking on history of substance abuse or psychosocial problems and prescription of the requested medication (oxycodone) or of any narcotic.

### Analytic approach

We used a mixed-method integration approach to converge the quantitative and qualitative data by analyzing the datasets separately and comparing results to merge findings.[21]

For quantitative data, we constructed multivariable logistic regression models to identify predictors of physicians stating “drug-seeking” as a potential diagnosis for the sciatica patient. Experimental design factors were included as predictors, with 2-way interactions included when significant. Other patient, physician, or practice variables significant at p<0.10 in bivariate analysis were included as potential predictors, with backwards selection used to fit final multivariate models. Chi-square and Fisher exact tests were used to compare other outcomes by presence/absence of a drug-seeking diagnosis and other design factors.

The diagnostic categories were open ended, and therefore were coded in two steps with direct input from clinical experts. First, raw data was exported and clinical experts developed broad diagnostic categories. Coding was conducted by the two analysts independently and QC was conducted on 20% of all coded data. A random sample of 20% of the data was selected for QC. During QC, a separate clinician consultant was given a list of the raw data and a coding list and asked to code the raw data. The clinician’s codes were compared to the study coders to identify discrepancies.

For the qualitative component, thematic content analysis[22] began with two analysts undertaking inductive “initial coding”[23] by independently coding a subset of transcripts and meeting to reach consensus. A codebook with initial codes and definitions captured whether the physician mentioned the possibility of drug seeking and if so the physician’s decision and reasoning regarding whether the patient was drug seeking. Analysts applied initial codes to another subset of transcripts, again meeting to reach consensus and revise the codebook as needed. As this process continued, analysts engaged in “focused coding”[23] by elaborating codes used more often. This process was repeated for ten batches of transcripts, at which point analysts agreed on the codebook and were applying it consistently. One analyst coded subsequent transcripts in batches; after each batch, analysts discussed and resolved questions.

One analyst(LW) conducted thematic analysis. Physicians were grouped according to whether they prescribed oxycodone as requested (based on responses in the quantitative interview data) and by patient characteristics. Frequencies of the most common codes were compared across groups, and quotations within groups were closely examined to fully develop themes and identify any substantive differences between groups.

## Results

### Quantitative analysis

Table 1 illustrates the distribution of “patient” characteristics in the filmed scenarios and the characteristics of the 192 physicians included in the study. Table 2 shows diagnosis rates by different characteristics of the sciatica “patients” and physicians. A diagnosis of drug seeking behavior was included by 20/96 physicians viewing the active request for oxycodone for symptoms consistent with sciatica(21%) and 3/96 physicians viewing the passive request for help with back pain(3%;p<0.001). Despite concerns about drug-seeking, physicians indicating a drug-seeking diagnosis were no less likely to prescribe the requested medication (2/23[9%] vs. 18/169[11%], p = 1.00 for oxycodone; 14/23[61%] vs. 102/169[60%], p = 0.96 for any narcotic). Physicians who included drug-seeking behavior as a diagnosis were more likely to ask about a history of substance abuse, though rates of seeking information about psychosocial problems were not different(Table 2). Physicians who viewed patients making an active request for oxycodone had eight-fold greater odds of indicating a drug-seeking diagnosis compared to physicians viewing the passive medication request(OR:8.10; 95% CI:2.11–31.15; p = 0.002; Table 3). Physicians who did not report receiving incentive payments as part of income were also more likely to indicate a drug-seeking diagnosis(OR:4.37; 95% CI:1.36–14.03; p = 0.013).

**Table 1:** Patient (simulated) and physician characteristics, N = 192.

	N (%) or Mean (SD)
**Patient**	
Age	~45
Sex^a^	
Male	96 (50%)
Female	96 (50%)
Race^a^	
White	64 (33%)
Black	64 (33%)
Hispanic	64 (33%)
SES^a^	
Lower	96 (50%)
Upper	96 (50%)
Medication Request^a^	
Active	96 (50%)
Passive	96 (50%)
**Physician Subjects**	
Age	49.4 (9.6)
Sex^a^	
Male	96 (50%)
Female	96 (50%)
Race	
White	106 (57.6%)
African American	14 (7.6%)
Asian	47 (25.5%)
Other	17 (9.2%)
Ethnicity	
Hispanic	11 (5.9%)
Not Hispanic	175 (94.1%)
Experience^a^	
<20 years	96 (50%)
>20 years	96 (50%)
Practice Type	
Family Practitioner	95 (49%)
Internist	86 (45%)
General Practitioner	11 (6%)
International Medical Graduate	
Yes	63 (34%)
No	123 (66%)

^a^ by balanced factorial study design

**Table 2:** Rates of drug-seeking diagnosis and associated actions for the sciatica “patient”.

	Levels	Drug-seeking diagnosis	
		n/N (%)	P-value*
Overall		23/192 (12.0%)	
**Characteristics:**			
Patient Gender	Female	9/96 (9.4%)	0.27
	Male	14/96 (14.6%)	
Patient Race	Black	5/64 (7.8%)	0.042
	Hispanic	5/64 (7.8%)	
	White	13/64 (20.3%)	
Patient SES	Lower	10/96 (10.4%)	0.50
	Upper	13/96 (13.5%)	
Medication Request Type	Active	20/96 (20.8%)	<0.001
	Passive	3/96 (3.1%)	
Physician Gender	Male	10/96 (10.4%)	0.50
	Female	13/96 (13.5%)	
Physician Experience	Less	11/96 (11.5%)	0.82
	More	12/96 (12.5%)	
**Actions taken:**			
Ask about substance abuse	No	16/167 (9.6%)	0.016
	Yes	7/25 (28.0%)	
Ask about psychosocial problems	No	21/171 (12.3%)	1.00
	Yes	2/21 (9.5%)	

* P-values are from chi-square tests or Fisher exact tests for sparse data.

**Table 3:** Final multivariable model predicting a diagnosis of drug-seeking for the sciatica “patient”.

	Odds ratio (95% CI)*	P-value
**Medication Request Type**		
Active	8.10 (2.11, 31.15)	0.002
Passive	Reference	
**Patient SES**		
Lower	1.09 (0.38, 3.10)	0.87
Upper	Reference	
**Patient Race**		
Black	0.28 (0.08, 0.97)	0.06
Hispanic	0.28 (0.07, 1.03)	
White	Reference	
**Patient Gender**		
Female	0.59 (0.21, 1.69)	0.32
Male	Reference	
**Physician Experience**		
Less	0.95 (0.34, 2.68)	0.92
More	Reference	
**Physician Gender**		
Male	0.54 (0.19, 1.56)	0.26
Female	Reference	
**What percent of your income is from incentive payments?**		
None	4.37 (1.36, 14.03)	0.013
> 1%	Reference	

* Other variables considered for inclusion in the multivariable model were: request information about substance abuse; amount of income earned through incentive payments; ever been named in a lawsuit; practice culture—business; and financial impact of a patient leaving the practice. None of these variables met the threshold to be included in the final model.

### Qualitative analysis

Evaluation of open-ended responses showed that, with a request for oxycodone, 55%(52/95) respondents mentioned the topic of drug seeking. Among physicians who viewed a patient requesting oxycodone and mentioned drug seeking(n = 52), only two(4%) decided that “drug seeking” was the most likely diagnosis, and neither of those prescribed a narcotic; both physicians were viewing white patients. Most physicians who considered drug-seeking behavior indicated that they were not certain about it or decided the patient was more likely not drug seeking(50/52, 96%), whether or not they prescribed a narcotic. Their detailed responses revealed several main lines of thought(Table 4), and thematic results were consistent across patient characteristics, including race/ethnicity(results not shown).

**Table 4:** Judgments about drug seeking by medication requested among physicians who mentioned drug seeking in the qualitative interview for sciatica patient (N = 52).

	Request for Oxycodone (N = 52)^a^	
	*Prescribed any narcotic*	
	Yes	No
	N = 37 (71%)	N = 15 (29%)
Decision- yes, drug seeking	0	2
		(13%)
Decision- no, unsure^b^	37	13
	(100%)	(87%)
• Rationale- appeared truthful	25/37	9/13
	(68%)	(69%)
• Rationale- how well knows patient	9/37	4/13
	(24%)	(31%)
• Rationale- benefit of the doubt	8/37	4/13
	(22%)	(31%)
• Rationale- gather more information	12/37	1/13
	(32%)	(8%)

^a^Qualitative interview missing for one respondent

^b^Some respondents provided more than one rationale

#### The story fits

The most common rationale for deciding against drug seeking was making a judgment that the patient appears truthful and “the story fits” (34/50,68%; Table 5). Physicians typically looked for clues in the patient’s symptom presentation, demeanor, and sometimes employment status to judge whether the patient was being truthful and expressing a real pain experience.

**Table 5:** Quotations supporting drug-seeking judgment rationales.

*The “story fits”/ patient truthful* “First of all, her just kind of tone and the description seemed to indicate the severity of her symptoms. She’s definitely miserable and it was going to affect her work ability.” “I felt his demeanor, his discussion of his symptoms, the statement that he was a vice president…Perhaps I’m gullible, but I believed his story.” “The guy sounds like he’s legitimate. He’s had a lot of pain. He hasn’t gotten over it. He’s even missed work.” “The patient is in obvious pain. I mean just looking at her….” “The fact that he specifically mentioned oxycodone, you know, you do have that…in the back of your mind. But, I think he was sincere and I believed what he said.”
*Knowing the patient* “And we all have that concern [drug seeking], but I think there are generally established patients that they trust us and we, in turn, have a sense of who that patient is.” “The first thing is obviously I know this patient for, let’s say, several years, and I never detected any suspicious specific requests for narcotic pain medications. That’s one issue.” “…I like to know more about this patient’s whole, his past medical score, what he has, social history, what he has used in terms of any narcotics or any illegal or steroid drugs, you know, and any history of alcoholism before I can really prescribe those narcotics.”
*Gather more information* “Acute back pain can be very painful, so I would give her a few tablets, not too much, until I get [a] diagnosis. …[If] all the tests are normal,…then I would advise her on the behavior change about not seeking drug..” “Yeah, I’d give him a three-day supply, maybe 12 pills, and then I would know with the MRI and my physical exam. …I can do a little socket test on them, I can do a Babinski on them…. So I’m going to be able to pick up clues that he doesn’t have what he doesn’t have.”
*Giving the patient the benefit of the doubt* “Every patient deserves the benefit of the doubt. This patient tried another medication… and it seemed to be effective. …If that can alleviate her pain and if that can improve her performance, her functionality, I would prescribe this medication, but I wouldn’t give her a large dose of this medication, nor would I give a large quantity of this medication.” (PCP prescribed narcotic) “But by the same token, no matter how skeptical I feel, you have to give the patient the benefit of the doubt…I’m going to treat the patient just like any other patient but with a little caution. With a pinch of salt.” (PCP did not prescribe narcotic)

#### Knowing the patient

The vignettes were described to subjects as a new complaint by an established patient, and some physicians mentioned the importance of knowing the patient and his/her history for making a decision about drug-seeking (13/50,26%; Table 5). This criterion of knowing the patient was raised both by physicians who did and did not prescribe a narcotic. Although knowing the patient well was an important consideration for some physicians, it was one factor and did not trigger an automatic granting of the requested medication. Physicians who assumed they knew the patient well and prescribed a narcotic also spoke of needing to believe that the symptoms were genuine.

#### Weighing the risks

Half of the physicians who mentioned drug-seeking described a process of weighing the risks of under-treating pain and medication abuse(25/50,50%). They recognized the potential for misuse of narcotics, but also were cognizant of the need to provide relief to patients experiencing pain. A physician provided a typical description of the dilemma:

“He could be having legitimate back pain, you know, so you hate to jump to conclusion and judge him, and you want to help him. But, then again, you don’t want to be too generous with your patient medication.”

Physicians spoke of two approaches for managing this dilemma:

Gather more information. Some physicians reserved judgment until they could gather more information(13/50,26%; Table 5). This approach was mentioned more commonly among those who prescribed a narcotic(12/37,32%) than those who did not(1/13,8%). Those who wanted to gather more information managed the risk of abuse by reducing the addictive potential (i.e., prescribing a short course or “lesser” narcotic) until more information was available. Other physicians spoke about gathering clues from the physical exam and interactions with the patient, which were not part of the experiment.

Give patients the benefit of the doubt. Physicians who did and did not prescribe a narcotic spoke about the importance of giving the patient the benefit of the doubt when initially presenting with pain(12/50,24%; Table 5). Those who prescribed a narcotic often said they would manage the risk of abuse by prescribing a short course of treatment or using a low dose, similar to physicians who would gather more information. Those who did not prescribe a narcotic often described a plan to attempt non-narcotic modalities first.

Some physicians directly connected the two approaches for weighing risk of abuse versus under-treatment.

“You know, give the guy a break, give him the benefit of the doubt. …You give him the medicines, and if he still pushes you—let’s say he won’t get the MRI but he still wants the morphine—you may have a problem patient on your hands, and you have to be aware of that.”

## Discussion

Assessment of pain challenges PCPs, who struggle to balance the need to treat pain adequately with concern about the risks of diversion or abuse of prescription pain medications.[24,25] This study represents one of the first systematic attempts to understand the thought processes that physicians use when evaluating patients initially complaining of pain and requesting pain medications. By combining quantitative and qualitative analyses in a mixed methods approach, we are able to statistically identify factors associated with physician suspicion of drug-seeking behavior by patients and understand physicians’ strategies for assessing whether a patient is drug seeking. The findings suggest directions for future interventions to improve the management of pain in outpatient settings.

When viewing a patient with pain who requested a narcotic pain medication by name, almost one-quarter of physicians listed drug-seeking behavior as a possible diagnosis, and over half at least considered drug-seeking behavior in their thought process. They rarely considered that possibility for patients making a more general request for pain relief. Although the direct request for a specific medication strongly increased physician suspicion, such patients were also more likely to be prescribed narcotics.[17] The qualitative analysis reveals that even when physicians consider drug-seeking they do not automatically decide that a patient is seeking drugs rather than experiencing real pain.

When designing the scenarios we had deliberately included only the generic drug name (oxycodone) out of concern that a request for a branded narcotic pain medication would generate excess suspicion in physicians. Accordingly, we cannot comment on whether the diagnoses of drug-seeking behavior or the decisions to prescribe narcotics would have differed with a request for a branded drug. The vignettes were not primarily designed to depict patients with drug-seeking behaviors, although one potential “red flag” was included in the form of the patient describing use of pain medication prescribed for someone else (their spouse).

The quantitative analyses showed that the specific medication request was by far the strongest predictor of a drug-seeking diagnosis, consistent with prior studies of non-opioid medications.[17,26] Physicians were slightly more likely to include drug-seeking as a diagnosis for white patients as compared to Black or Hispanic patients. This result differs from prior research suggesting that clinicians more often assume drug-seeking behaviors by patients from minority groups.[27–32] The qualitative data did not reveal substantive differences. Physicians who considered drug seeking as a diagnosis were also more likely to ask patients about substance abuse history, a reasonable concern, but were not more likely to assume that the patient had a history of psychosocial problems. Taken together, these associations do not suggest a significant element of stigma or disparities in these assessments, though we cannot exclude the possibility of social desirability bias affecting the physician responses in a setting when their responses were being recorded.

In the qualitative analyses, physicians reported three main strategies. Many physicians described making a judgment during the visit about the patient’s truthfulness in their descriptions. The context for the sciatica vignette was an established patient returning for a new complaint, and physicians also indicated the importance of their prior knowledge of the patient and relationships developed over time, reflecting the importance of the doctor-patient relationship in primary care and especially in the evaluation of challenging complaints and conditions.

Physicians also described approaches that they would take to simultaneously begin managing the patient’s pain and further assess whether drug seeking was an issue. These included the use of narcotic pain medications in short course or at low doses; making the assumption that the risk of harm would be relatively small and that narcotics would not be continued or increased. Other physicians proposed the initial use of non-narcotic medications, or assessment of patient compliance with other steps (such as imaging or physical therapy). Inherent in these approaches is the idea that patients who progress through these steps and continue to request use of narcotic pain medications would be more likely to have genuine pain and be less likely to be seeking the medications for secondary gain.

Although new guidelines have recently been published for the management of chronic pain,[33] to date there has been limited research on the assessment and management of initial presentations with pain or potential drug-seeking behavior by patients or of approaches for reducing the risks of narcotic pain medications.[34] Accordingly, our findings suggest important areas that could be explored to understand how patients begin using opioid pain medications. Approaches proposed by the physicians responding to this vignette, including the use of initial therapy and testing to determine the severity of the pain complaint and the risk of drug seeking, should be evaluated in future research. Likewise, many physicians stated that they would ask more questions to assess whether patients were drug seeking; development and validation of instruments that could be used in primary-care settings would address this need.[35]

There are limitations that must be kept in mind when assessing these findings. The use of video vignettes may threaten external validity. To address this concern, we developed clinically authentic scenarios, with clinicians involved in scripting and filming. The physicians viewed the vignettes during a practice day in their offices; 96% found the patient presentation very or reasonably typical of patients seen in their everyday practice. While many physicians identified drug-seeking behavior as a possibility, overall level of certainty about this assessment was relatively low and physicians concerned about drug-seeking were equally likely to prescribe narcotic medications as those who did not state such a concern. Vignettes including requests for the brand-name version of a narcotic painkiller might be more likely to trigger concerns for drug-seeking.

These limitations are counterbalanced by important strengths of this analysis. The experimental design allows for unconfounded estimates of the impact of patient requests on physician decision-making. The video vignettes allow for more detailed presentation than possible with written scenarios, while providing a consistency of presentation that would be difficult to attain with standardized patients. By employing a mixed-methods approach we are able to move beyond simple description of clinical decisions and gain insight into the underlying cognitive reasoning processes.

## Conclusions

Although drug-seeking behavior is increasingly recognized as a potential problem, there is no established definition nor universally accepted standard of management, so we cannot comment on whether the level of concern expressed by physicians in this study was appropriate, too high, or too low. Some guidelines for managing patients requesting pain medications have been proposed,[24,25,34,36,37] but they have not yet been widely taken up by practitioners. Our findings provide important insights into how primary care clinicians approach decisions about drug-seeking related to narcotic pain medications and highlight the need for further work in this clinical area and for the development of tools to help clinicians work together with patients to manage pain without overusing potentially harmful medications.

## References

[1] Gureje O, Simon GE, Von Korff M (2001) A cross-national study of the course of persistent pain in primary care. Pain 92: 195–200. 11323140 10.1016/s0304-3959(00)00483-8

[2] Mantyselka P, Kumpaso E, Ahonen R, Kumpusalo A, Kauhanen J, Viinamaki H, et al (2001) Pain as a reason to visit the doctor: a study in Finnish primary health care. Pain 89: 175–180. 11166473 10.1016/s0304-3959(00)00361-4

[3] Reid MC E-H L.L.; Weber M.B.; Kerns R.D.; Rogers E.L.; O'Connor P.G. (2002) Use of Opioid Medication for Chronic Noncancer Pain Syndromes in Primary Care. J Gen Intern Med 17: 173–179. doi: 10.1046/j.1525-1497.2002.10435.x 11929502 10.1046/j.1525-1497.2002.10435.x PMC1495018

[4] Gureje O, Von Korff M, Simon GE, Gater R (1998) Persistent pain and well-being: a World Health Organization Study in Primary Care. JAMA 280: 147–151. 9669787 10.1001/jama.280.2.147

[5] American Pain Society Quality of Care Committee (1995) Quality improvement guidelines for the treatment of acute pain and cancer pain. JAMA 274: 1874–1880. 7500539 10.1001/jama.1995.03530230060032

[6] Department of Veterans Affairs: National Pain Management Coordinating Committee (2000) Pain as the 5th Vital Sign Toolkit.

[7] Kirsch B, Berdine H, Zablotsky D (2000) Management strategy: identifying pain as the fifth vital sign. VHSJ: 49–59.

[8] Prevention CfDCa (2012) CDC Grand Rounds: Prescription Drug Overdoses—A U.S. Epidemic. Morbidity and Mortality Weekly Report. 22237030

[9] Jones CM, Mack KA, Paulozzi LJ (2013) Pharmaceutical overdose deaths, United States, 2010. JAMA 309: 657–659. doi: 10.1001/jama.2013.272 23423407 10.1001/jama.2013.272

[10] Prevention CfDCa (2013) Policy Impact: Prescription Painkiller Overdoses. Injury Prevention & Control.

[11] Prevention CFDCa (2013) Press Release: Opioids drive continued increase in drug overdose deaths.

[12] Bohnert AS, Valenstein M, Bair MJ, Ganoczy D, McCarthy JF, Ilgen MA, et al (2011) Association between opioid prescribing patterns and opioid overdose-related deaths. JAMA 305: 1315–1321. doi: 10.1001/jama.2011.370 21467284 10.1001/jama.2011.370

[13] Hall AJ, Logan JE, Toblin RL, Kaplan JA, Kraner JC, Bixler D, et al (2008) Patterns of abuse among unintentional pharmaceutical overdose fatalities. JAMA 300: 2613–2620. doi: 10.1001/jama.2008.802 19066381 10.1001/jama.2008.802

[14] Fishbain DA, Cole B, Lewis J, Rosomoff HL, Rosomoff RS (2008) What percentage of chronic nonmalignant pain patients exposed to chronic opioid analgesic therapy develop abuse/addiction and/or aberrant drug-related behaviors? A structured evidence-based review. Pain Med 9: 444–459. doi: 10.1111/j.1526-4637.2007.00370.x 18489635 10.1111/j.1526-4637.2007.00370.x

[15] Logan J, Liu Y, Paulozzi L, Zhang K, Jones C (2013) Opioid prescribing in emergency departments: the prevalence of potentially inappropriate prescribing and misuse. Med Care 51: 646–653. doi: 10.1097/MLR.0b013e318293c2c0 23632597 10.1097/MLR.0b013e318293c2c0

[16] Grover CA, Close RJ, Wiele ED, Villarreal K, Goldman LM (2012) Quantifying drug-seeking behavior: a case control study. J Emerg Med 42: 15–21. doi: 10.1016/j.jemermed.2011.05.065 21958455 10.1016/j.jemermed.2011.05.065

[17] McKinlay JB, Trachtenberg F, Marceau LD, Katz JN, Fischer MA (2014) Effects of patient medication requests on physician prescribing behavior: results of a factorial experiment. Medical Care in press. 10.1097/MLR.0000000000000096 PMC4151257 24848203

[18] Maserejian NN, Fischer MA, Trachtenberg FL, Yu J, Marceau LD, McKinlay JB, et al (2014) Variations among primary care physicians in exercise advice, imaging, and analgesics for musculoskeletal pain: results from a factorial experiment. Arthritis Care Res (Hoboken) 66: 147–156. 24376249 10.1002/acr.22143 PMC4067704

[19] Marceau L, McKinlay J, Piccolo R (2012) Clinical vignettes in health services research: advantages and limitations of different formats. AcademyHealth Orlando, FL.

[20] Konrad TR, Link CL, Shackelton RJ, Marceau LD, von dem Knesebeck O, Siegrist J, et al (2010) It's about time: physicians' perceptions of time constraints in primary care medical practice in three national healthcare systems. Med Care 48: 95–100. doi: 10.1097/MLR.0b013e3181c12e6a 20057331 10.1097/MLR.0b013e3181c12e6a PMC3621071

[21] Fetters MD, Curry LA, Creswell JW (2013) Achieving Integration in Mixed Methods Designs-Principles and Practices. Health Services Research 48: 2134–2156. doi: 10.1111/1475-6773.12117 24279835 10.1111/1475-6773.12117 PMC4097839

[22] Green J, Thorogood N (2009) Qualitative methods for health research. Thousand Oaks, CA: Sage.

[23] Lofland J, Lofland LH (1995) Analyzing Social Settings: A Guide to Qualitative Observation and Analysis. Belmont, CA: Wadsworth.

[24] Chou R, Fanciullo GJ, Fine PG, Adler JA, Ballantyne JC, Davies P, et al (2009) Clinical Guidelines for the Use of Chronic Opioid Therapy in Chronic Noncancer Pain. The Journal of Pain 10: 113–130. doi: 10.1016/j.jpain.2008.10.008 19187889 10.1016/j.jpain.2008.10.008 PMC4043401

[25] Streltzer J, Ziegler P, Johnson B, American Academy of Addiction P (2009) Cautionary guidelines for the use of opioids in chronic pain. Am J Addict 18: 1–4. doi: 10.1080/10550490802544508 19219659 10.1080/10550490802544508

[26] Kravitz RL, Epstein RM, Feldman MD, et al (2005) Influence of patients’ requests for direct-to-consumer advertised antidepressants: A randomized controlled trial. JAMA 293: 1995–2002. doi: 10.1001/jama.293.16.1995 15855433 10.1001/jama.293.16.1995 PMC3155410

[27] Bonham VL (2001) Race, ethnicity, and pain treatment: striving to understand the causes and solutions to the disparities in pain treatment. J Law Med Ethics 29: 52–68. 11521272 10.1111/j.1748-720x.2001.tb00039.x

[28] Committee on Understanding and Eliminating Racial and Ethnic Disparities in Health Care IoM (2009) Unequal Treatment: Confronting Racial and Ethnic Disparities in Health Care: The National Academic Press 737 p.

[29] Mossey JM (2011) Defining racial and ethnic disparities in pain management. Clin Orthop Relat Res 469: 1859–1870. doi: 10.1007/s11999-011-1770-9 21249483 10.1007/s11999-011-1770-9 PMC3111792

[30] Pletcher MJ, Kertesz SG, Kohn MA, Gonzales R (2008) Trends in opioid prescribing by race/ethnicity for patients seeking care in US emergency departments. JAMA 299: 70–78. doi: 10.1001/jama.2007.64 18167408 10.1001/jama.2007.64

[31] Shavers VL, Bakos A, Sheppard VB (2010) Race, ethnicity, and pain among the U.S. adult population. J Health Care Poor Underserved 21: 177–220. doi: 10.1353/hpu.0.0255 20173263 10.1353/hpu.0.0255

[32] Burgess DJ, Crowley-Matoka M, Phelan S, Dovidio JF, Kerns R, Roth C, et al (2008) Patient race and physicians' decisions to prescribe opioids for chronic low back pain. Soc Sci Med 67: 1852–1860. doi: 10.1016/j.socscimed.2008.09.009 18926612 10.1016/j.socscimed.2008.09.009

[33] Dowell D, Haegerich TM, Chou R (2016) Cdc guideline for prescribing opioids for chronic pain—united states, 2016. JAMA 315: 1624–1645. doi: 10.1001/jama.2016.1464 26977696 10.1001/jama.2016.1464 PMC6390846

[34] Beletsky L, Rich JD, Walley AY (2012) Prevention of fatal opioid overdose. JAMA 308: 1863–1864. doi: 10.1001/jama.2012.14205 23150005 10.1001/jama.2012.14205 PMC3551246

[35] Krebs EE, Bergman AA, Coffing JM, Campbell SR, Frankel RM, Matthias MS (2014) Barriers to guideline-concordant opioid management in primary care—a qualitative study. J Pain 15: 1148–1155. doi: 10.1016/j.jpain.2014.08.006 25179150 10.1016/j.jpain.2014.08.006

[36] Chou R (2009) 2009 Clinical Guidelines from the American Pain Society and the American Academy of Pain Medicine on the use of chronic opioid therapy in chronic noncancer pain: what are the key messages for clinical practice? Pol Arch Med Wewn 119: 469–477. 19776687

[37] McCarberg BH (2011) Pain management in primary care: strategies to mitigate opioid misuse, abuse, and diversion. Postgrad Med 123: 119–130. 10.3810/pgm.2011.03.2270 21474900

